# Supplementary material for: Evidence for Adaptive Selection in the Mitogenome of a Mesoparasitic Monogenean Flatworm Enterogyrus malmbergi
Source: Genes (Basel). 2019 Oct 30;10(11):863. doi: 10.3390/genes10110863 (PMC6896049; doi:10.3390/genes10110863)
Supplement: Supplementary file 1 [file genes-10-00863-s001.zip › Supplementary Figure S1.pdf]

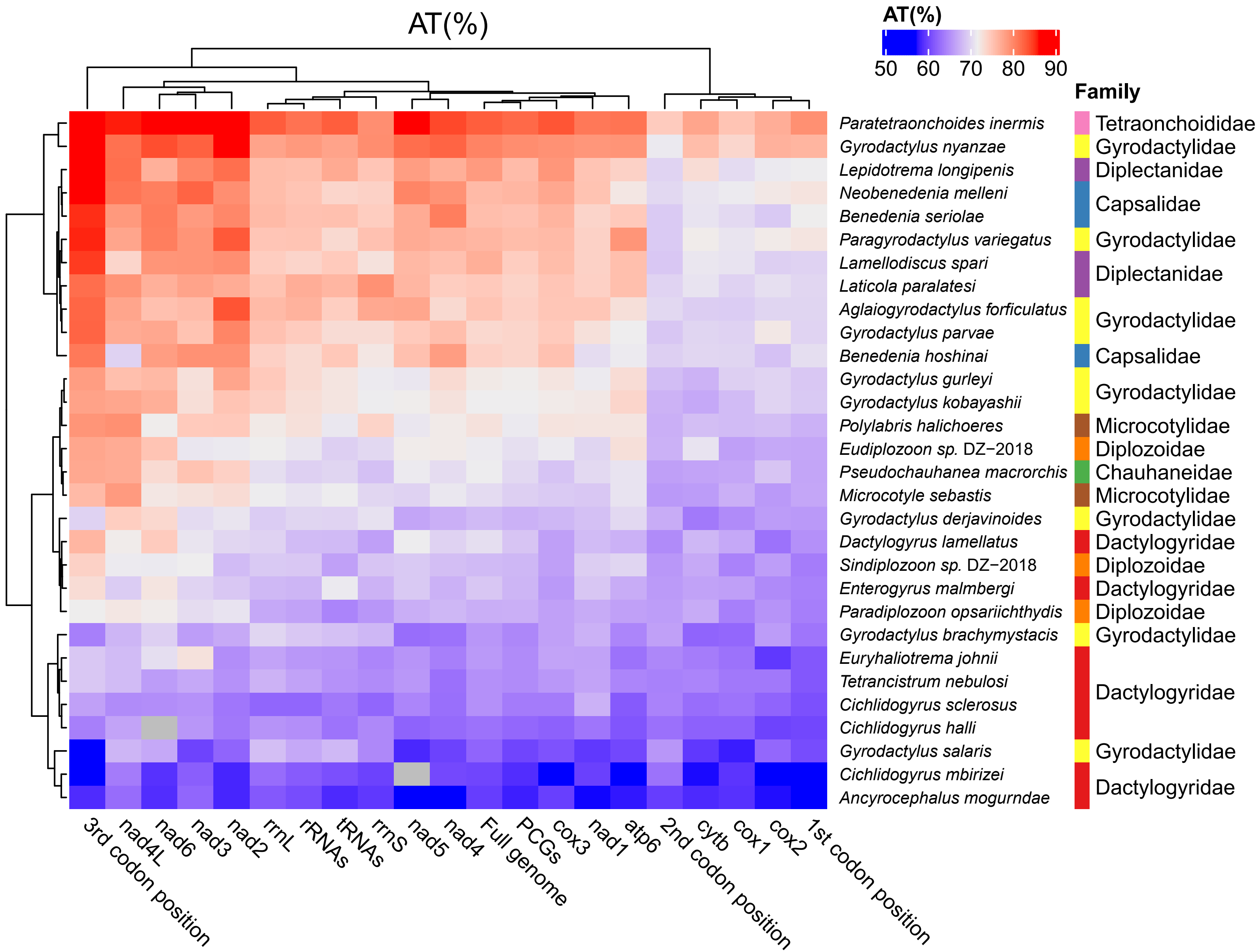
AT(%)
AT(%)
50
60
70
80
90
Family
Paratetraonchoides inermis
Gyrodactylus nyanzae
Lepidotrema longipenis
Neobenedenia melleni
Benedenia seriolae
Paragyrodactylus variegatus
Lamellodiscus spari
Laticola paralatesi
Aglaiogyrodactylus forficulatus
Gyrodactylus parvae
Benedenia hoshinai
Gyrodactylus gurleyi
Gyrodactylus kobayashii
Polylabris halichoeres
Eudiplozoon sp. DZ−2018
Pseudochauhanea macrorchis
Microcotyle sebastis
Gyrodactylus derjavinoides
Dactylogyrus lamellatus
Sindiplozoon sp. DZ−2018
Enterogyrus malmbergi
Paradiplozoon opsariichthydis
Gyrodactylus brachymystacis
Euryhaliotrema johnii
Tetrancistrum nebulosi
Cichlidogyrus sclerosus
Cichlidogyrus halli
Gyrodactylus salaris
Cichlidogyrus mbirizei
Ancyrocephalus mogurndae
Tetraonchoididae
Gyrodactylidae
Diplectanidae
Capsalidae
Gyrodactylidae
Diplectanidae
Gyrodactylidae
Capsalidae
Gyrodactylidae
Microcotylidae
Diplozoidae
Chauhaneidae
Microcotylidae
Gyrodactylidae
Dactylogyridae
Diplozoidae
Dactylogyridae
Diplozoidae
Gyrodactylidae
Dactylogyridae
Gyrodactylidae
Dactylogyridae
3rd codon position
nad4L
nad6
nad3
nad2
rrnL
rRNAs
tRNAs
rrnS
nad5
nad4
Full genome
PCGs
cox3
nad1
atp6
2nd codon position
cytb
cox1
cox2
1st codon position
