# Supplementary material for: Evidence for Adaptive Selection in the Mitogenome of a Mesoparasitic Monogenean Flatworm Enterogyrus malmbergi
Source: Genes (Basel). 2019 Oct 30;10(11):863. doi: 10.3390/genes10110863 (PMC6896049; doi:10.3390/genes10110863)
Supplement: Supplementary file 1 [file genes-10-00863-s001.zip › Supplementary Table S1.docx]

| **Fragment** | **Gene or region** | **Primer name** | **Sequence (5’-3’)** | **Length (bp)** |
| --- | --- | --- | --- | --- |
| ***Ancyrocephalus mogurndae*** | | | |  |
| F1 | *COX1* | HLF1 | GGKTGGACTTTYTAGGCCAG | 927 |
|  |  | HLR1 | CAHACTCGACGNGGTAAACCGC |  |
| F2 | *COX1-16S* | HLF2 | TACCAGGGTAGTAATCTC | 622 |
|  |  | HLR2 | CGACTGACGTATCACAAACC |  |
| F3 | *16S* | HLF3 | GTTACCTTTTGCCTCATGAT | 842 |
|  |  | HLR3 | HCGGTCTTWACTCAACTCAT |  |
| F4 | *16S-12S* | HLF4 | GTTGCTACCTCGATGTTGAC | 549 |
|  |  | HLR4 | CTAATCTGCGTCGTTAATCGG |  |
| F5 | *12S* | HLF5 | CAGTGCCAGCAYCCNCGGTTA | 487 |
|  |  | HLR5 | GATTGACGKGCGDTGTGTAC |  |
| F6 | *12S-COX2* | HLF6 | GGTGTAAGTTAATAGATTCGG | 572 |
|  |  | HLR6 | CTAGCAAGTTAGTAGGAGC |  |
| F7 | *COX2* | HLF7 | TTGGTCGTCAGTGRTACTGR | 202 |
|  |  | HLR7 | ATACGBCCTGGNATCGCATC |  |
| F8 | *COX2-ND5* | HLF8 | GCTAGTAACCTCCTCGGATG | 1938 |
|  |  | HLR8 | TCAAGCAAAGCAGGGACAGC |  |
| F9 | *ND5-COX3* | HLF9 | CGGAAGTGTTGAATGTGCTG | 2857 |
|  |  | HLR9 | GTATAGTGGTAGTGCATTCCC |  |
| F10 | *COX3* | HLF10 | GADATTCCACTWTTNGGTTG | 337 |
|  |  | HLR10 | CCACAWAGTGYCAGTATCA |  |
| F11 | *COX3-CYTB* | HLF11 | GGACTTATAACTTGGTTG | 594 |
|  |  | HLR11 | CCCAATAGGACATCTGGTG |  |
| F12 | *CYTB* | HLF12 | CAYATGGGACGAGCYTTATA | 526 |
|  |  | HLR12 | CTGGCTTAATGGCMGMAGGAG |  |
| F13 | *CYTB-ND1* | HLF13 | TCTGTTTGCTTAGCATTG | 3794 |
|  |  | HLR13 | CGATACTGCACCAGTGAGCTC |  |
| F14 | *ND1* | HLF14 | CGHAAGGGVCCCAAWAAGGYRGG | 700 |
|  |  | HLR14 | CATAGCGCACNCGAGGYARTG |  |
| F15 | *ND1-COX1* | HLF15 | CCTGCCTTTTTGCTTGTGAG | 1575 |
|  |  | HLR15 | GAGATAGCGGATTGTATGGTG |  |
| ***Enterogyrus malmbergi*** | | | |  |
| F1 | *COX1-16S* | C6F1 | CATCAGCAGAAAGAATGCATATG | *1939* |
|  |  | C6R1 | CAAGTCAACATCGAGGTAGC |  |
| F2 | *16S* | C6F2 | GACGGAAAGACCCCVAGAGY | 277 |
|  |  | C6R2 | AGATAAGAACCGACCTGGCT |  |
| F3 | *16S-12S* | C6F3 | GAGCTATTTGGTGCAGCAGC | 520 |
|  |  | C6R3 | CTAATCTACATCACATAAACAAG |  |
| F4 | *12S* | C6F4 | CACAGTGCCAGCANCCRCGG | 529 |
|  |  | C6R4 | CTACCATGTTACGACTTATC |  |
| F5 | *12S-COX2* | C6F5 | GGACTTGTTAGTAAAAGGAAG | 568 |
|  |  | C6R5 | GAAGACGTTACTAACATACGC |  |
| F6 | *COX2* | C6F6 | AAGGTTDTTGGNCGTCAATG | 284 |
|  |  | C6R6 | TGTCNCGYACCHCACAACTC |  |
| F7 | *COX2-CYTB* | C6F7 | GTAGATGCAATTCCAGGTCG | 4860 |
|  |  | C6R7 | CAACATGAGCTGCAAACACAC |  |
| F8 | *CYTB* | C6F8 | GTCTTHTTGKGCTGCAACAG | 438 |
|  |  | C6R8 | GATCGARGCATAGCATAWTA |  |
| F9 | *CYTB-ND1* | C6F9 | GTACACAGCATGCTTAGGCTTG | 3924 |
|  |  | C6R9 | CTAGTAATTATCAAAAACCAC |  |
| F10 | *ND1* | C6F10 | CGTAAGRGGCKAAACAAGGTAGG | 705 |
|  |  | C6R10 | ATCAWAACGAACNCGAGGTA |  |
| F11 | *ND1-COX1* | C6F11 | CATTATGGCGTGGTTTAGAGC | 1599 |
|  |  | C6R11 | ACGATCAAACAACAACATAG |  |
